# A qualitative exploration of the lived experience of informal caregivers of people with severe mental illness and co-existing long-term conditions

**DOI:** 10.1192/j.eurpsy.2024.211

**Published:** 2024-08-27

**Authors:** C. Carswell, J. V. E. Brown, D. Shiers, P. Coventry, N. Siddiqi

**Affiliations:** ^1^Department of Health Sciences, University of York, York; ^2^Psychosis Research Unit, Greater Manchester Mental Health NHS Trust; ^3^Division of Psychology and Mental Health, University of Manchester, Manchester; ^4^Primary Care and Health Sciences, Keele University, Keele; ^5^York Environmental Sustainability Institute, University of York; ^6^Centre for Health and Population Sciences, Hull York Medical School, York, United Kingdom

## Abstract

**Introduction:**

People with severe mental illness (SMI), including schizophrenia and bipolar disorder, experience significant health inequalities and are more likely to develop long-term physical health conditions (LTCs), such as type 2 diabetes and cardiovascular disease. Many people with SMI rely on informal caregivers, typically friends and family, to support their health and enable them to live in the community. Informal caregivers of people with SMI experience high levels of caregiver burden, social isolation, and poor health outcomes. However, it is unclear how co-existing LTCs contribute to the caregiving experience.

**Objectives:**

The aim of this study was to explore the lived experience of informal caregivers of people with co-existing SMI and LTCs.

**Methods:**

We conducted a qualitative study with informal caregivers of people with co-existing SMI and LTCs in England. We recruited 12 informal caregivers and conducted five semi-structured interviews and two focus groups between December 2018 and April 2019. The interviews and focus groups were audio recorded, transcribed verbatim and thematically analysed.

**Results:**

SMI impacts profoundly on the health and well-being of both service users and their informal caregivers. Service users were described as too unwell with their SMI to engage in self-management of their mental and physical health, with the primary responsibility for these tasks falling to informal caregivers. There were significant barriers to adequate physical healthcare for service users, therefore informal caregivers needed to advocate extensively for their loved ones to ensure access to services. Informal caregivers felt significantly under-supported and struggled with the caregiver burden associated with SMI and LTCs. This burden included the constant monitoring of risk, anxiety around the vulnerability of their loved one, repeated hospitalisations, physical health concerns, lack of respite services, lack of recognition of their role, the guilt associated with paternalistic care, shame and stigma, and the difficulties managing the changeable nature of SMI.

**Conclusions:**

Informal caregivers of people with SMI face an additional caregiver burden resulting from co-existing LTCS. This adds substantially to their caring role, yet they do not receive the necessary support, and therefore their own health and wellbeing are negatively impacted. Improved recognition of the role of informal caregivers and additional support, including improved provision of respite services, are needed to improve the well-being of informal caregivers.

**Disclosure of Interest:**

C. Carswell: None Declared, J. Brown: None Declared, D. Shiers Consultant of: DS is an expert adviser to the National Institute for Health and Care Excellence Centre for Guidelines; the views expressed are those of the authors and not those of National Institute for Health and Care Excellence., P. Coventry: None Declared, N. Siddiqi: None Declared

